# The patterns of admixture, divergence, and ancestry of African cattle populations determined from genome-wide SNP data

**DOI:** 10.1186/s12864-020-07270-x

**Published:** 2020-12-07

**Authors:** N. Z. Gebrehiwot, E. M. Strucken, H. Aliloo, K. Marshall, J. P. Gibson

**Affiliations:** 1 Centre for Genetic Analysis and Applications, School of Environmental and Rural Science, University of New England, Armidale, NSW 2351 Australia; 2 International Livestock Research Institute and Centre for Tropical Livestock Genetics and Health, Nairobi, Kenya

**Keywords:** Admixture, African crossbreds, African indigenous, *Bos taurus*, *Bos indicus*, Effective population size, Genetic differentiation, Linkage disequilibrium, SNPs

## Abstract

**Background:**

Humpless *Bos taurus* cattle are one of the earliest domestic cattle in Africa, followed by the arrival of humped *Bos indicus* cattle. The diverse indigenous cattle breeds of Africa are derived from these migrations, with most appearing to be hybrids between *Bos taurus* and *Bos indicus*. The present study examines the patterns of admixture, diversity, and relationships among African cattle breeds.

**Methods:**

Data for ~ 40 k SNPs was obtained from previous projects for 4089 animals representing 35 African indigenous, 6 European *Bos taurus*, 4 *Bos indicus,* and 5 African crossbred cattle populations. Genetic diversity and population structure were assessed using principal component analyses (PCA), admixture analyses, and Wright’s *F* statistic. The linkage disequilibrium and effective population size (*Ne*) were estimated for the pure cattle populations.

**Results:**

The first two principal components differentiated *Bos indicus* from European *Bos taurus*, and African *Bos taurus* from other breeds. PCA and admixture analyses showed that, except for recently admixed cattle, all indigenous breeds are either pure African *Bos taurus* or admixtures of African *Bos taurus* and *Bos indicus.* The African zebu breeds had highest proportions of *Bos indicus* ancestry ranging from 70 to 90% or 60 to 75%, depending on the admixture model. Other indigenous breeds that were not 100% African *Bos taurus*, ranged from 42 to 70% or 23 to 61% *Bos indicus* ancestry. The African *Bos taurus* populations showed substantial genetic diversity, and other indigenous breeds show evidence of having more than one African taurine ancestor. *Ne* estimates based on *r*^*2*^ and *r*^2^_adj_ showed a decline in *Ne* from a large population at 2000 generations ago, which is surprising for the indigenous breeds given the expected increase in cattle populations over that period and the lack of structured breeding programs.

**Conclusion:**

African indigenous cattle breeds have a large genetic diversity and are either pure African *Bos taurus* or admixtures of African *Bos taurus* and *Bos indicus.* This provides a rich resource of potentially valuable genetic variation, particularly for adaptation traits, and to support conservation programs. It also provides challenges for the development of genomic assays and tools for use in African populations.

**Supplementary Information:**

The online version contains supplementary material available at 10.1186/s12864-020-07270-x.

## Background

Based on skeletal evidence, Sahara rock art, and Egyptian dynastic representations, the humpless taurine cattle (*Bos taurus)* are thought to be the earliest domestic cattle in Africa [1]. Archaeological evidence suggested that African cattle were domesticated in the eastern Sahara 10,000 to 8000 years before present (BP) by hunter-gatherers [2]. But genetic evidence suggests a single domestication event in the Near East and subsequent crossing with wild aurochs in the southern Fertile Crescent and/or North Africa [3]. Using genome-wide SNP data of 67 ancient Near Eastern *Bos taurus* and modern populations, Verdugo et al. [4] suggested that the ancient Levantine genome affinity with Moroccan aurochs implies that the distinct phenotypes and genotypes in African *Bos taurus* cattle may stem from roots in the southern Fertile Crescent. In their review of the evidence, Stock and Gifford-Gonzalez [5] concluded that *Bos taurus* cattle likely spread across the Sinai and into the Nile Delta 7000 to 8000 BP, then across North Africa, and subsequently into the Nile and the then-grassy Sahara, possibly with additional inputs through the Horn of Africa. Humpless, presumably *Bos taurus* cattle first appear to be present south of the Sahara about 4500 to 4000 BP [6, 7]. A few depictions of Egyptian cattle show humped animals, which are claimed as evidence for the presence of *Bos indicus* cattle in Egypt from 3500 BP [8]. The earliest evidence for *Bos indicus* cattle in sub-Saharan Africa is in East Africa, where all samples, that could be analyzed from two sites dated around 2000 to 2500 BP, were of *Bos indicus* or Sanga (a hybrid of *Bos indicus* and *Bos taurus*) type [9]. This suggests that *Bos indicus* genes were already predominant in the pastoral systems in this region. Payne and Hodges [6] concluded that *Bos taurus* cattle, however, remained predominant in Ethiopia and East Africa until recently despite many waves of *Bos indicus* introductions to the region from about 2500 BP onwards.

Currently, Africa is home to more than 180 cattle breeds or distinct cattle populations [10], and several authors have made classifications of present-day African indigenous breeds of cattle. Rege and Tawah [11] suggested four categories of indigenous breeds: *Bos taurus*, *Bos indicus* (zebu), Sanga (*Bos taurus* × *Bos indicus* hybrid)*,* and Zenga (Sanga×zebu hybrid). According to Lenstra and Bradley [12], African *Bos taurus* breeds are those that have short ears and no hump, while zebu breeds are those that have long floppy ears and a prominent hump. Subsequent results based on molecular marker data [13] and results presented here show that the genetic diversity of African cattle is more complex than this, most particularly, no African indigenous breeds have been shown to be pure *Bos indicus*. Thus, the term “zebu”, as applied to African cattle breeds, means that the breed has a hump, but it does not imply that the breed is pure *Bos indicus*, despite much of the literature using zebu and *Bos indicus* as synonymous when applied to African cattle.

Studies of mitochondrial DNA (mtDNA) variation indicated that the two major groups of cattle, *Bos taurus* and *Bos indicus,* were genetically distinct before domestication [14–16]. A PCA result by Verdugo et al. [4] using genome sequence data on ancient cattle samples revealed that cattle origins consisted of two divergent aurochs populations that formed the basis of the *Bos indicus* and *Bos taurus* divide. These authors also showed, using mtDNA sequence data, that there was male-driven *Bos indicus* introgression into the Near East *Bos taurus* populations. Studies of microsatellite DNA and Y-chromosomal markers showed extensive introgression of male *Bos indicus* genes into existing African cattle populations [17–19], all of which currently carry *Bos taurus* mtDNA, indicating male-driven introgression of *Bos indicus* genes into the previously *Bos taurus* African cattle populations. Based on genome-wide autosomal SNP markers, Weerasinghe et al. [13] showed that all indigenous cattle breeds from Tanzania, Kenya, Uganda, and Ethiopia were admixtures of *Bos indicus* and African *Bos taurus.*

The present study provides one of the most extensive analyses of the genetic diversity of African cattle breeds based on genome-wide SNP data to date. We undertook admixture and principal component analyses, Wright’s *F* statistic (*F*_*ST*_ and *F*_*IS*_), and linkage disequilibrium (LD) analyses to obtain a clear picture of the patterns of admixture and genetic diversity of African indigenous and crossbred populations and to compare their diversity to exotic breeds.

## Results

### Principal components and admixture analyses of indigenous breeds

**Fig. 1:**
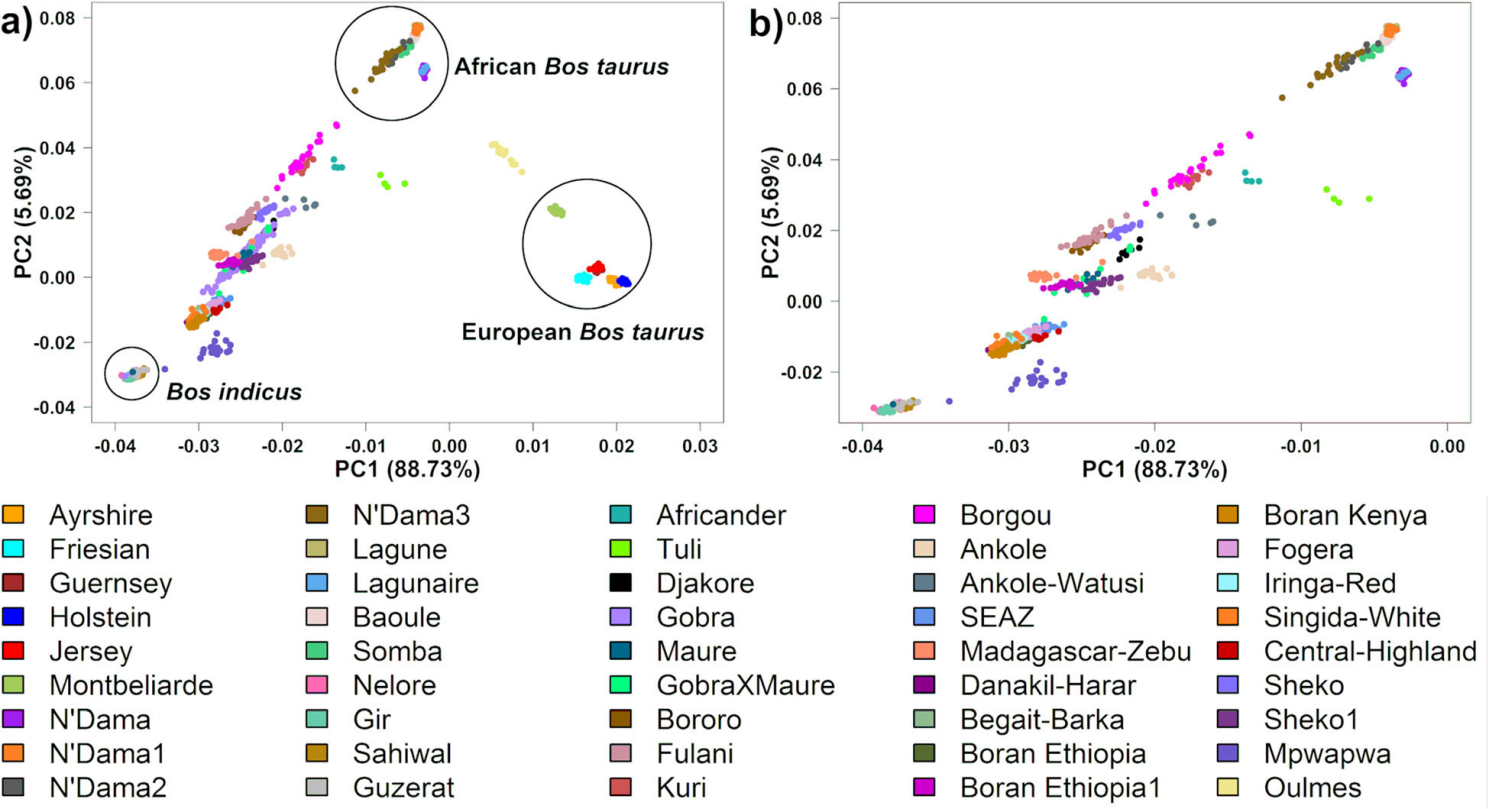
PC1 vs. PC2 when using the whole dataset. **a** Showing all African indigenous and reference breeds. **b** A magnified plot of (**a**) showing African samples with Gobra removed

Principal component analyses were performed to explore and visualize the genetic variation between different breeds and to identify potential sub-structures within the data. The first five principal components (PC) obtained from an analysis of all indigenous and crossbred cattle populations from East and West Africa, and including African and European taurine reference breeds as well as indicine reference breeds, explained a total of 96.1% of the variation in the genomic relationship matrix (GRM). The first two components accounted for 88.7 and 5.7% of the total genetic variation, respectively, and differentiated the *Bos indicus,* European *Bos taurus,* and African *Bos taurus* breeds from each other as the apexes of a triangle in the plot area (Fig. 1a). The indicine reference breeds, Nelore, Gir, Sahiwal, and Guzerat, grouped tightly together while the African taurine populations clustered in two distinct groups (Figs. 1a, b, and S1a). The first African taurine group comprised N’Dama (from Guinea) and Lagunaire, and the second group included N’Dama1 (from Cote d’Ivoire), N’Dama2 (from Southeast Burkina Faso), N’Dama3 (from Southwest Burkina Faso), Lagune, Baoule and Somba. N’Dama2 and especially N’Dama3 appeared to include animals that spread towards the pooled *Bos indicus* reference breeds, showing that they are not pure African taurine breeds (Fig. 1a, b), and, therefore, these breeds were excluded from the African taurine reference breeds in later Admixture analyses.

A separate PCA was performed to evaluate in more detail the genetic structure among the eight African taurine reference populations (Figure S2). The first, second, and third PCs explained 32.1, 20.5, and 7% of the total variance among the African taurine breeds, respectively. Somba and Baoule clustered tightly together, while all other samples formed separate single clusters, except N’Dama3, which split into two clusters (for more detailed results, see Gebrehiwot et al. [20]).

The majority of the East African indigenous breeds that are classified as zebu breeds (Danakil-Harar, Begait-Barka, Ethiopian Boran, Fogera, Iringa-Red, Singida-White, Kenyan Boran, Central Highland, and SEAZ), clustered together on or slightly to the right and at the indicine end of the axis between indicine and the first African taurine group (N’Dama and Lagunaire, Fig. 1b). Note that in Fig. 1b, the Gobra sample has been removed as it is not a pure breed sample, and it obscured the position of other samples in the plot. The Sheko1, Maure, Boran Ethiopia1, and Madagascar-zebu clustered further towards the Africa taurine breeds (i.e., lower *Bos indicus* admixture) and spread between the two axes that connect the indicine to the first African taurine group (axis 1) versus the second African taurine group (axis 2). Most of the hybrid animals between Gobra and Maure (Gobra x Maure) sit in this second cluster, aligning with axis 1. The Madagascar-zebu is distinct from all the zebu breeds being the only zebu breed that sits on axis 2.

The Ankole, Djakore, and Sheko (Sanga breeds), and Bororo and Fulani (zebu breeds) form the third cluster located more towards the African taurine breeds, with Ankole and Djakore close to axis 1 and the other breeds on or slightly to the left of axis 2 (Fig. 1b). The Bororo (also known as Red Fulani) and Fulani clustered together. Gobra showed a large genetic diversity along axis 1 (Fig. 1a). The Borgou and Kuri lie on axis 2, and the Ankole-Watusi and Africander lie on axis 1, all more towards African taurine than other breeds. The Tuli forms an outlier group consistent with high African taurine ancestry but well to the right of axis 1 indicating admixture with European taurine. Except for one outlier, the composite dual-purpose Mpwapwa breed clustered at the indicine end but well to the right of axis 1. The Moroccan Oulmes Zaer clustered in an intermediary position between African and European taurine breeds.

**Fig. 2:**
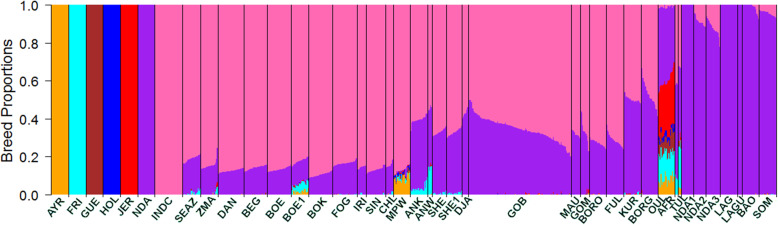
Breed proportion of indigenous African breeds from a supervised (*K* = 7) Admixture analysis. AYR = Ayrshire, FRI = Friesian, GUE = Guernsey, HOL = Holstein, JER = Jersey, NDA = N’Dama, INDC = Indicine, SEAZ = Small East African Zebu, ZMA = Madagascar-zebu, DAN = Danakil-Harar, BEG = Begait-Barka, BOE = Boran-Ethiopia, BOE1 = Boran-Ethiopia1, BOK = Boran-Kenya, FOG = Fogera, IRI = Iringa-Red, SIN = Singida-White, CHL = Central Highland, MPW = Mpwapwa, ANK = Ankole, ANW = Ankole-Watusi, SHE = Sheko, SHE1 = Sheko1, DJA = Djakore, GOB = Gobra, MAU = Maure, GOM = Gobra x Maure, BORO = Bororo, FUL = Fulani, KUR = Kuri, BORG = Borgou, OUL = Oulmes Zaer, AFR = Africander, TUL = Tuli, NDA1 = N’Dama1, NDA2 = N’Dama2, NDA3 = N’Dama3, LAG = Lagune, LAGU = Lagunaire, BAO = Baoule, SOM = Somba

**Fig. 3:**
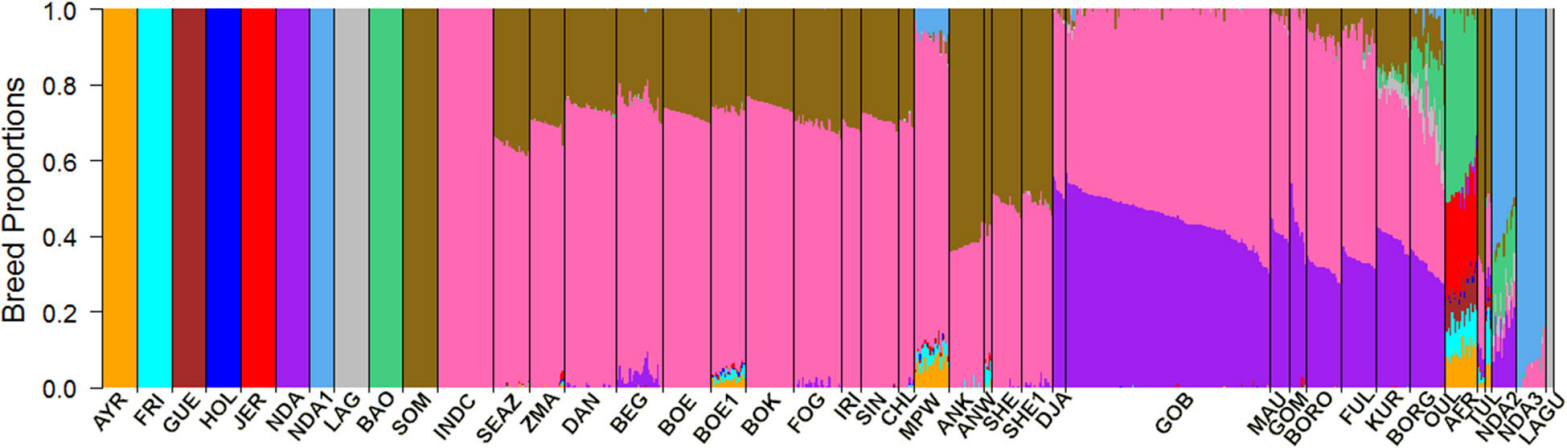
Breed proportion of indigenous African breeds from a supervised (*K* = 11) Admixture analysis. AYR = Ayrshire, FRI = Friesian, GUE = Guernsey, HOL = Holstein, JER = Jersey, NDA = N’Dama, NDA1 = N’Dama1, LAG = Lagune, BAO = Baoule, SOM = Somba, INDC = Indicine, SEAZ = Small East African Zebu, ZMA = Madagascar-zebu, DAN = Danakil-Harar, BEG = Begait-Barka, BOE = Boran Ethiopia, BOE1 = Boran Ethiopia1, BOK = Boran Kenya, FOG = Fogera, IRI = Iringa-Red, SIN = Singida-White, CHL = Central Highland, MPW = Mpwapwaa, ANK = Ankole, ANW = Ankole-Watusi, SHE = Sheko, SHE1 = Sheko1, DJA = Djakore, GOB = Gobra, MAU = Maure, GOM = Gobra x Maure, BORO = Bororo, FUL = Fulani, KUR = Kuri, BORG = Borgou, OUL = Oulmes Zaer, AFR = Africander, TUL = Tuli, NDA2 = N’Dama2, DNA3 = N’Dama3, LAGU = Lagunaire

Figures 2 and 3 illustrate the estimated breed ancestries from supervised Admixture Models 1 and 2 with *K* = 7 and *K* = 11, respectively. In Model 1, only one African taurine breed (N’Dama) was used together with a pooled indicine sample and five European taurine reference breeds. Model 2 included an additional four African taurine reference breeds to differentiate the African *Bos taurus* background. Consistent with the PCA, all African indigenous breeds, other than the pure African taurine breeds, were estimated to be an admixture of indicine and African taurine ancestries. Some breeds also showed small admixture with European taurine. Absolute estimates of ancestral proportions differed substantially between Admixture Model 1 versus Model 2, with Model 2 giving lower estimates of indicine ancestry. However, the ranking of breeds for indicine ancestry proportion was very similar between Model 1 and Model 2, and the following results summarised here are for Model 1. Overall, the indicine proportion was lower in West and South African breeds compared to East African breeds. However, the West African breeds, especially from Senegal, showed a wide range of *Bos indicus* ancestry. For example, the indicine component in Gobra ranged from 48.5 to 79.8% (average 65.3%), from 64.8 to 70.3% (average 67.8%) in Maure, and from 56.0 to 77.3% (average 66.2%) in Gobra x Maure crosses (Table 1).

**Table 1:** Admixture proportions from supervised analyses (mean ± SD) of African indigenous breeds for indicine, African taurine and total European taurine ancestry

Breed	*K* = 7			*K* = 11				
	Indicine	N’Dama	EUT	Indicine	N’Dama	Somba	EUT	NDA1 + LAG+BAO
Baoule	0.01 ± 0.02	0.99 ± 0.02	0	Fixed ancestral breeds				
Somba	0.04 ± 0.01	0.95 ± 0.01	0					
Lagune	0	0.99 ± 0.00	0					
N’Dama1	0	0.99 ± 0.00	0					
N’Dama2	0.08 ± 0.03	0.93 ± 0.03	0	0.04 ± 0.02	0.15 ± 0.05	0.02 ± 0.02	0	0.79 ± 0.12
N’Dama3	0.12 ± 0.04	0.88 ± 0.04	0	0.05 ± 0.04	0	0	0	0.95 ± 0.06
Lagunaire	0	0.99 ± 0.00	0	0	0	0	0	0.99 ± 0.00
Africander	0.42 ± 0.01	0.48 ± 0.02	0.10 ± 0.02	0.23 ± 0.01	0.06 ± 0.01	0.67 ± 0.02	0.04 ± 0.03	0
Tuli	0.32 ± 0.02	0.36 ± 0.02	0.32 ± 0.04	0.18 ± 0.02	0.06 ± 0.02	0.49 ± 0.01	0.26 ± 0.07	0.01 ± 0.01
Djakore	0.57 ± 0.02	0.43 ± 0.02	0	0.46 ± 0.02	0.52 ± 0.02	0.01 ± 0.01	0	0
Gobra	0.65 ± 0.06	0.35 ± 0.06	0	0.55 ± 0.06	0.45 ± 0.06	0.00 ± 0.01	0	0
Maure	0.68 ± 0.02	0.32 ± 0.02	0	0.57 ± 0.02	0.41 ± 0.02	0.02 ± 0.02	0	0
GobraxMaure	0.66 ± 0.06	0.33 ± 0.07	0.01 ± 0.01	0.56 ± 0.06	0.43 ± 0.07	0.01 ± 0.01	0.01 ± 0.01	0
Bororo	0.73 ± 0.02	0.27 ± 0.02	0	0.61 ± 0.02	0.31 ± 0.02	0.08 ± 0.02	0	0
Fulani	0.71 ± 0.03	0.29 ± 0.03	0	0.60 ± 0.03	0.34 ± 0.02	0.06 ± 0.02	0	0.01 ± 0.01
Kuri	0.50 ± 0.02	0.50 ± 0.02	0	0.38 ± 0.02	0.40 ± 0.02	0.17 ± 0.02	0	0
Borgou	0.47 ± 0.07	0.53 ± 0.07	0	0.37 ± 0.06	0.32 ± 0.03	0.10 ± 0.02	0	0.21 ± 0.01
Ankole	0.61 ± 0.02	0.36 ± 0.02	0.03 ± 0.02	0.37 ± 0.02	0	0.62 ± 0.02	0.00 ± 0.01	0
Ankole-Watusi	0.55 ± 0.02	0.32 ± 0.02	0.13 ± 0.04	0.35 ± 0.02	0	0.59 ± 0.02	0.06 ± 0.05	0
SEAZ	0.81 ± 0.02	0.18 ± 0.01	0.01 ± 0.01	0.64 ± 0.02	0	0.36 ± 0.02	0	0
Madegascar-zebu	0.85 ± 0.03	0.15 ± 0.01	0.01 ± 0.02	0.69 ± 0.03	0	0.31 ± 0.02	0.01 ± 0.02	0
Danakil-Harar	0.88 ± 0.01	0.12 ± 0.01	0	0.74 ± 0.01	0	0.26 ± 0.01	0	0
Begait-Barka	0.85 ± 0.04	0.14 ± 0.04	0.01 ± 0.04	0.72 ± 0.03	0.03 ± 0.02	0.24 ± 0.03	0.01 ± 0.01	0.00 ± 0.01
Boran Ethiopia	0.87 ± 0.01	0.13 ± 0.01	0	0.72 ± 0.01	0	0.28 ± 0.01	0	0
Boran Ethiopia1	0.83 ± 0.02	0.12 ± 0.01	0.06 ± 0.01	0.69 ± 0.01	0	0.26 ± 0.01	0.05 ± 0.01	0
Boran Kenya	0.90 ± 0.01	0.10 ± 0.01	0	0.75 ± 0.01	0	0.25 ± 0.01	0	0
Fogera	0.84 ± 0.01	0.16 ± 0.01	0	0.69 ± 0.01	0	0.30 ± 0.02	0.00 ± 0.02	0
Iringa-Red	0.85 ± 0.03	0.14 ± 0.01	0.01 ± 0.03	0.68 ± 0.02	0	0.31 ± 0.01	0.01 ± 0.02	0
Singida-White	0.87 ± 0.01	0.13 ± 0.01	0	0.71 ± 0.02	0	0.29 ± 0.01	0	0
Central Highland	0.85 ± 0.13	0.15 ± 0.01	0.00 ± 0.01	0.70 ± 0.02	0	0.30 ± 0.012	0	0
Sheko	0.67 ± 0.02	0.32 ± 0.01	0.01 ± 0.01	0.48 ± 0.02	0.00 ± 0.01	0.51 ± 0.02	0	0
Sheko1	0.67 ± 0.02	0.32 ± 0.01	0.01 ± 0.01	0.49 ± 0.02	0.01 ± 0.01	0.50 ± 0.02	0	0
Mpwapwa	0.87 ± 0.03	0.01 ± 0.02	0.12 ± 0.03	0.81 ± 0.03	0	0.01 ± 0.02	0.11 ± 0.05	0.07 ± 0.02
Oulmes Zaer	0.01 ± 0.01	0.38 ± 0.04	0.60 ± 0.04	0	0.02 ± 0.03	0	0.52 ± 0.09	0.45 ± 0.05

*EUT* 5 European *Bos taurus breeds*, *NDA1* N’Dama1, *LAG* Lagune, *BAO* Baoule

In East Africa, the Ankole, Ankole-Watusi, Sheko, and Sheko1 showed the lowest indicine proportions ranging from 55.3 to 67.7% (Fig. 2 and Table 1). Ankole-Watusi, Ankole, and Ethiopian Boran1 showed average exotic breed proportions larger than 1%. Ankole-Watusi had 13% exotic taurine ancestry, which was attributed mainly to Friesians and Ayrshires based on Model 1. The South African Tuli and Africander had low indicine ancestry with high levels of exotic taurine ancestry (32 and 10%, respectively; Fig. 1, Table 1).

The Oulmes Zaer were almost exclusively of taurine ancestry with 38.8% African and 60% European taurine ancestry. The synthetic Mpwapwa breed had European taurine (12%) and indicine (87%) ancestry (Fig. 2, Table 1). The African taurine breeds N’Dama1, Lagune, Lagunaire, and Baoule showed > 99% reference N’Dama ancestry, whereas N’Dama2 and N’Dama3 (sampled from Southeast and Southwest Burkina Faso, respectively), and Somba showed a high indicine ancestry (8.4, 11.8 and 4.4% indicine, respectively) (Fig. 2, Table 1).

Admixture Model 2, which included five African *Bos taurus* breeds as ancestral reference breeds, identified a difference in the assigned African taurine ancestry between cattle breeds from East, South, and West Africa. The East and South African breeds had a Somba background predominantly. Begait-Barka was the only East African breed with more than 1% N’Dama ancestry. The two South African breeds, Africander and Tuli, showed 6% N’Dama ancestry, while the West African indigenous breeds had N’Dama background (Fig. 3, Table 1) predominantly. However, Bororo, Fulani, Kuri, and Borgou also showed some Somba ancestry (7.8, 6.2, 16.7, and 9.9%, respectively), and the latter two also showed a Lagune background of 3.5 and 6.1%, respectively (Fig. 3, Table 1). Borgou showed an additional N’Dama1 content of 2.4%.

Under Model 2, the African taurine proportion of Oulmes Zaer was 45% Lagune and 2% N’Dama ancestry. The European breed proportion of Mpwapwa remained almost unchanged, but the indicine content was reduced, and African taurine content of 7% N’Dama1 and 1% Somba was detected (Fig. 3, Table 1). Of the African taurine breeds that were not used as reference ancestral breeds, N’Dama2 appeared to be an admixture of all reference African taurine breeds (N’Dama1 = 58.8%, Baoule = 15.7%, N’Dama = 15.3%, Somba = 2.1%, and Lagune = 4.0%) plus 4.1% *Bos indicus* ancestry. N’Dama3 showed 94.2% N’Dama1 plus 5.2% *Bos indicus* ancestry. The Lagunaire appeared as 100% Lagune (Fig. 3, Table 1).

### Admixture and principal components analyses of crossbred cattle

Principal components and Admixture analyses were conducted, including East (Kenya, Uganda, Ethiopia, and Tanzania) and West (Senegal) African crossbred animals. Admixture Model 3 with *K* = 12 extended Model 2 by adding Montbeliarde as a reference breed due to its reported use in crossbreeding in Senegal [21].

**Fig. 4:**
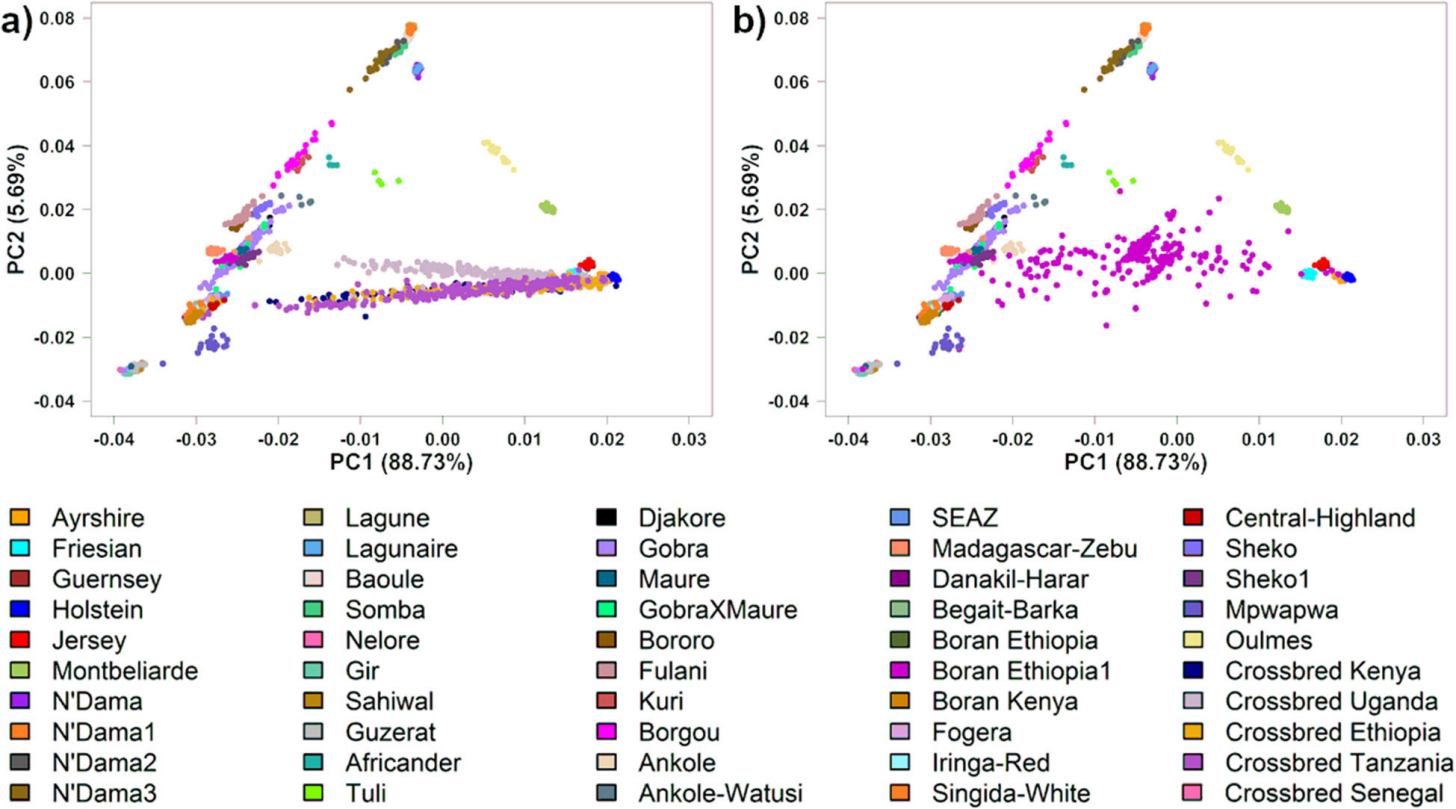
PC1 vs. PC2 plot for African indigenous, crossbred, and reference breeds. **a** East African crossbreds. **b** Senegal crossbreds

Figure 4 shows the PC plots for the same analyses as in Fig. 1 but with crossbred animals added to the plot. The crossbreds from Ethiopia, Kenya, and Tanzania were distributed between the East African zebu and European dairy breeds, while the crossbred animals from Uganda were located between the Ugandan Sanga breed (Ankole) and the European dairy breeds (Fig. 4a). The Senegal crossbred animals exhibited a much greater genetic diversity with a much wider range of both indigenous and exotic dairy breed ancestries compared to the East African crossbreds (Fig. 4b).

**Fig. 5:**
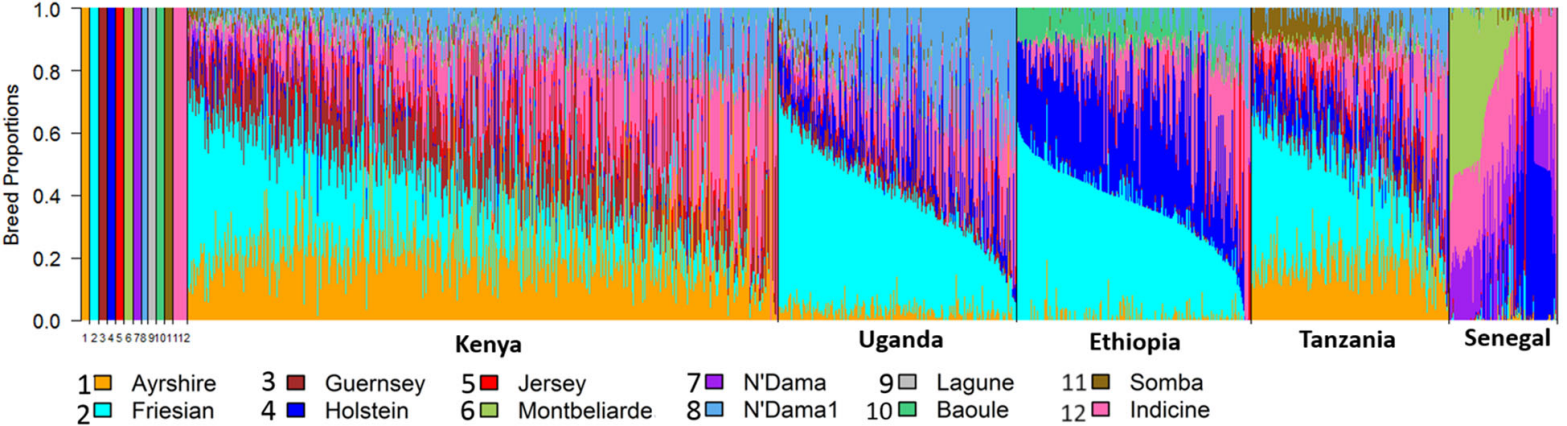
Breed proportion of crossbred cattle from a supervised (*K* = 12) Admixture analysis

With Admixture Model 3, the crossbred animals from Kenya showed an average exotic dairy proportion of 69%, mainly from Friesian (23%), followed by Ayrshire (23%), and Guernsey (16%). The Ugandan crossbreds showed an average exotic dairy proportion of 62% with the main contribution from Friesian (38%) and Holstein (14%). The Ethiopian and Tanzanian crossbred animals showed an average of 72% exotic dairy proportion; Ethiopian crossbreds had 36% Friesian and 30% Holstein, and Tanzanian crossbreds had 31% Friesian, 19% Ayrshire, and 12% Holstein (Fig. 5, Table 2). The Senegal crossbreds had an average exotic dairy proportion of 50%, ranging from almost 0 to 98%, and the primary contributions coming from Montbeliarde (22%) and Holstein (12%) (Fig. 5, Table 2).

**Table 2:** Estimates (±SD) of individual and total exotic dairy breed proportions for African crossbred cows

Breed	Total dairy proportion	Ayrshire	Friesian	Guernsey	Holstein	Jersey	Montbeliarde
Kenyan Crossbred	0.688 ± 0.202	0.228 ± 0.127	0.230 ± 0.126	0.157 ± 0.084	0.030 ± 0.091	0.036 ± 0.043	0.008 ± 0.009
Ugandan Crossbred	0.622 ± 0.195	0.033 ± 0.041	0.381 ± 0.141	0.048 ± 0.052	0.137 ± 0.132	0.015 ± 0.032	0.007 ± 0.009
Ethiopian Crossbred	0.721 ± 0.190	0.014 ± 0.024	0.360 ± 0133	0.004 ± 0.008	0.300 ± 0.139	0.036 ± 0.109	0.006 ± 0.009
Tanzanian Crossbred	0.719 ± 0.172	0.188 ± 0.092	0.307 ± 0.122	0.046 ± 0.042	0.121 ± 0.095	0.046 ± 0.032	0.011 ± 0.014
Senegalese Crossbred	0.503 ± 0.187	0.006 ± 0.016	0.016 ± 0.037	0.008 ± 0.018	0.222 ± 0.255	0.026 ± 0.103	0.224 ± 0.227

### Genetic relatedness and differentiation

Inbreeding, as represented by the *F*_*IS*_ value, was close to zero (between − 0.006 to 0.009) for most breeds across all breed groups (Table S1). The highest positive *F*_*IS*_ value of 0.049 was observed for Somba. The strongest negative *F*_*IS*_ was observed for N’Dama3 (− 0.109).

**Fig. 6:**
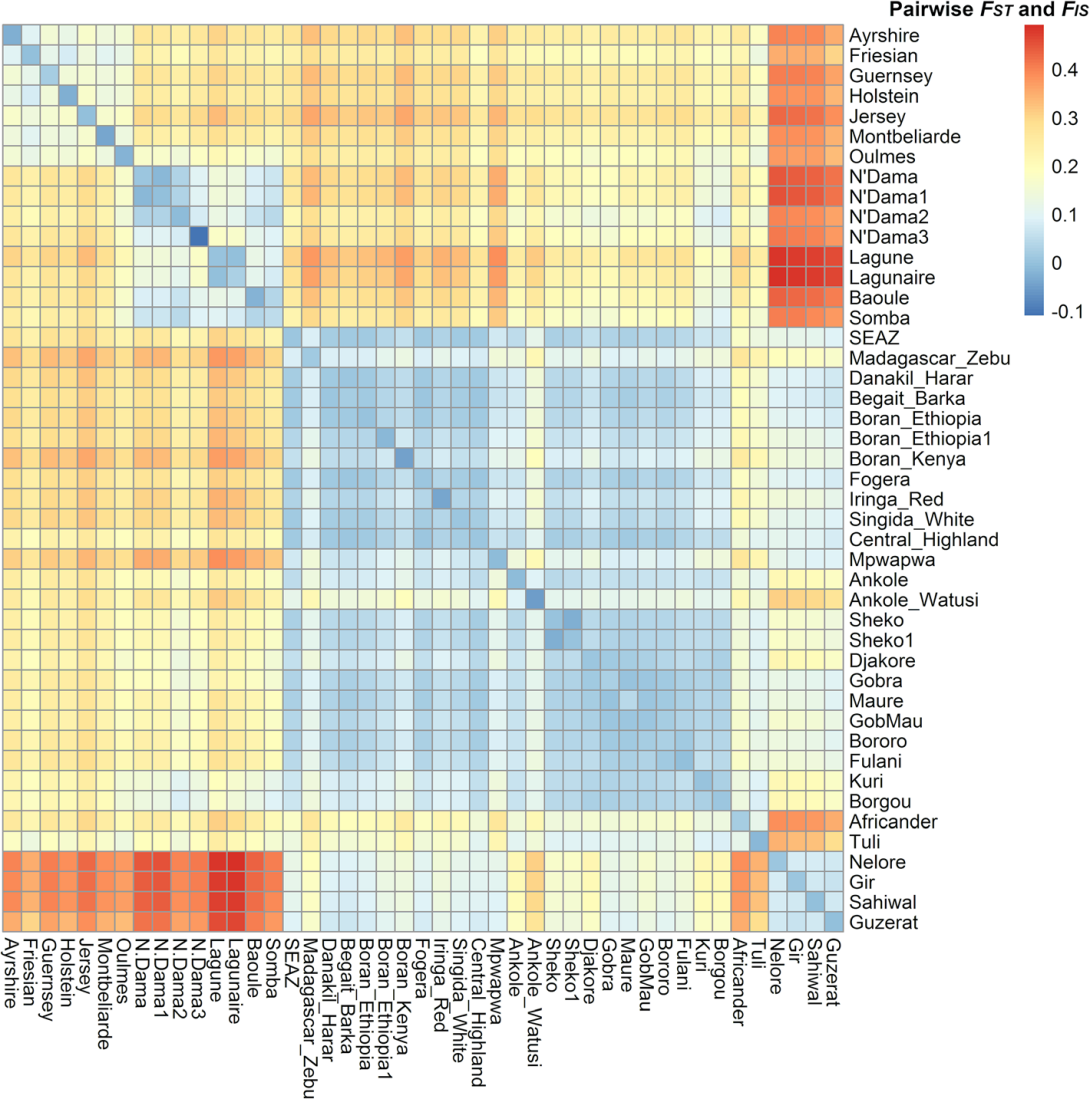
Heatmap of the relationship within and among different cattle breeds based on Weir and Cockerham pairwise *F*_*ST*_ values (off-diagonal) and Nei *F*_*IS*_ values (diagonal) as shown by the color scale bar on the right. Each cell represents a group of individuals of the same breeds

Breed differentiation, as represented by *F*_*ST*_ values, showed a strong divergence within different groups of breeds (European *Bos taurus*, African *Bos taurus*, zebu types, Sanga types including admixed breeds, and *Bos indicus*; Fig. 6, Table S1). Ranked from lowest to highest genetic differentiation between breeds within groups are zebu types, *Bos indicus*, Sanga types, African *Bos taurus,* and lastly, European *Bos taurus*. Some notable outliers within the breed groups are N’Dama3, which has high *F*_*ST*_ with all other Africa *Bos taurus* breeds; Madagascar-zebu with high *F*_*ST*_ values with all other zebu type breeds; the South African Africander and Tuli both have high *F*_*ST*_ with Sanga type breeds; and Ankole-Watusi which has a relatively high *F*_*ST*_ with other Sanga breeds.

### Extent and decay of linkage disequilibrium

**Fig. 7:**
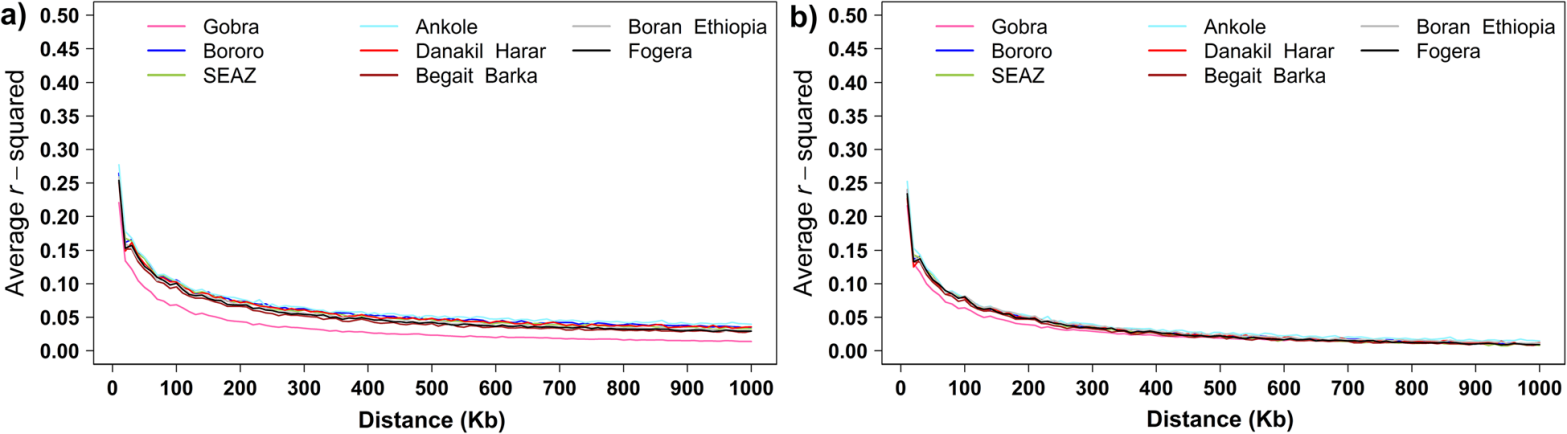
The decline of *r*^*2*^ (**a**) and *r*^2^_adj_ (**b**) with physical distance (Kb) in nine African breeds

The decay of squared correlations (*r*^2^) and adjusted squared correlation (*r*^2^_adj_) between phased alleles of pairwise SNP loci over increasing genome distances is illustrated in Fig. 7a and b, respectively, for the nine African indigenous breeds that had more than 20 animals after removing highly related animals from the data. Ankole had higher *r*^2^ and a lower rate of *r*^2^ decay, and Gobra showed lower *r*^2^ and a higher rate of *r*^2^ decay than the other populations across all distances (Fig. 7a), which translates into the lowest and highest estimates of *Ne* across all times, respectively.

### Past effective population size before and after adjusting *r*^2^ for sample size

**Fig. 8:**
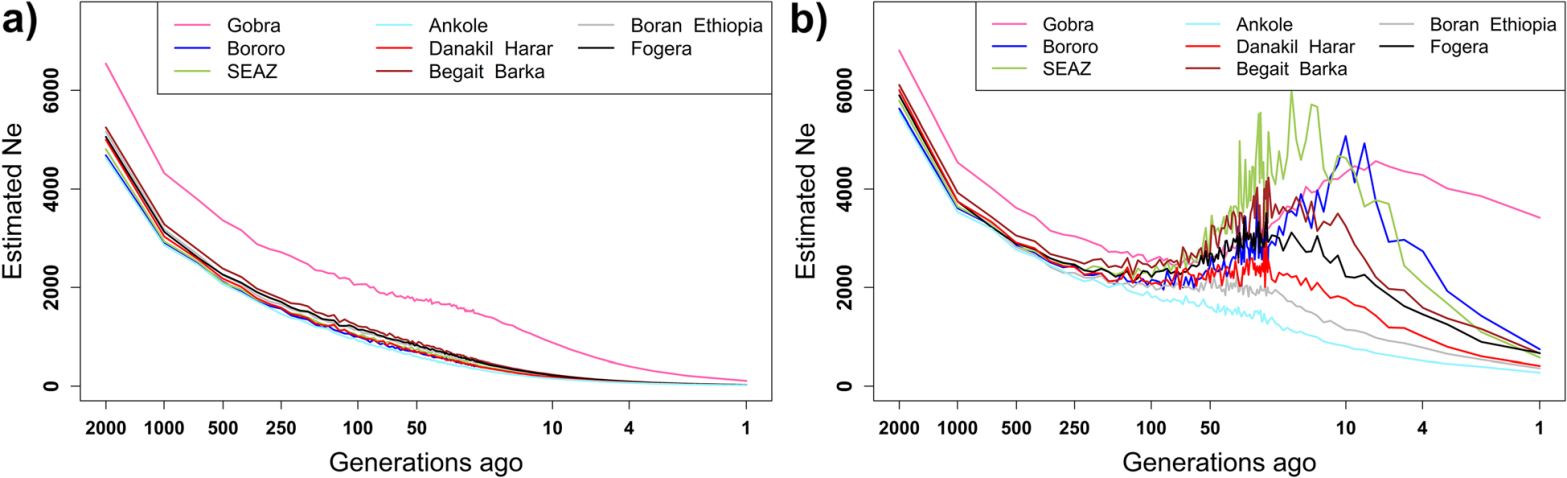
Effective population size over past generations (log-scaled) for African indigenous breeds using (**a**) *r*^2^ and (**b**) *r*^2^_adj_

*Ne* was calculated for various generations in the past using *r*^2^ and *r*^2^_adj_ for the nine African indigenous breeds in the analyses. *Ne* estimates using *r*^2^ declined steadily over time for all breeds (Fig. 8a). Except for Ankole, which showed a steady decline across all periods, the *Ne* estimates using *r*^2^_adj_ declined until around 200 generations ago and then held steady or increased markedly until 30 to 5 generations ago before declining again.

Gobra showed the highest and Ankole the lowest *Ne* at all generations using *r*^2^, with 107 and 18 at 1 generation ago, and 6544 and 4633 at 2000 generations ago, respectively. Similarly, estimates of *Ne* based on *r*^2^_adj_ for Gobra were highest at generation 1 and 2000, with 3418 and 6809, respectively, while the lowest *Ne* was found for Ankole, with 272 at generation 1 and 5557 at generation 2000. Estimated *Ne* using *r*^2^ for Bororo, SEAZ, Danakil-Harar, Fogera, Boran Ethiopia, and Begait-Barka at 1 and 2000 generations ago were 19 and 4687, 19 and 4812, 20 and 5005, 24 and 5063, 25 and 5168, 25 and 5255, respectively, while estimates of *Ne* using *r*^2^_adj_ were 743 and 5630, 576 and 5790, 410 and 6006, 665 and 5899, 363 and 5964, and 659 and 6109, respectively. Thus, across the nine breeds, the finite sampling adjustment to *r*^*2*^ increased *Ne* 14.8 to 39.2 fold at generation 1 and 1 to 1.2 fold at 2000 generations ago.

## Discussion

### Genetic diversity and relationships

Depending on the used data and underlying assumption about biological clocks, estimates of divergence between *Bos taurus* and *Bos indicus* vary from approximately 200,000 to 300,000 years BP [3, 14, 16, 22, 23], to 575,000 to 800,000 years BP [15, 24], to 2 m years BP [25]. Based on microsatellite data, MacHugh et al. [19] estimated the separation of the African and European taurine clades to be between 180,000 to 250,000 years ago, while the same group estimated divergence time at between 22,000 and 26,000 years ago using mtDNA [15]. Both estimates pre-date domestication.

The PCA and *F*_*ST*_ results showed a clear divergence between *Bos indicus*, African *Bos taurus*, and European *Bos taurus* reference breeds, which is in agreement with several previous studies [4, 26–32]. While the pattern of PCA results is expected to reflect in part the choice of SNPs on the assay, we have found very similar patterns for those breeds that have Illumina 777 k SNP data, despite the widely different pattern of breed allele frequency distributions of the SNPs on the 777 k versus the 50 k assays [33]. As shown here and in other studies, the first two principal components differentiate the groups with the largest genetic differences (PC1 *Bos taurus* vs. *Bos indicus*, PC2 European *Bos taurus* vs. Africa *Bos taurus*). Later principale components could be able to tease out smaller genetic differences, such as dairy vs beef breeds, however, coressponding beef reference breeds were not included. Additionally, some beef breeds are closer related to dairy breeds than other beef breeds or vice versa [34–36] which can make the choice of appropriate reference breeds cumbersome. As we were interested in the origin of African cattle and not their economic type, we chose to only include reference breeds that are historically proven to have been introduced in the regions under study.

Among the African cattle populations, there are no pure *Bos indicus* populations in our sample. Based on the range of samples included in our study, it is unlikely that any indigenous breeds of Africa are pure *Bos indicus.* As outlined in the background section, it has been assumed in the literature that the admixed indigenous breeds of Africa arose from *Bos indicus* cattle entering Africa and breeding with existing African *Bos taurus* populations [8, 9]. Verdugo et al. [4] concluded that *Bos taurus* populations in the Near East became admixed with *Bos indicus* likely due to human migrations around 4200 years BP. The populations they sampled in the Levant, which were the closest samples to the putative first route of *Bos indicus* into Africa through Egypt, showed a *Bos taurus* genotype that was closest to modern African *Bos taurus*. Therefore, it is possible that the first humped cattle entering Africa around 4000 years BP [6] may already have been hybrids between *Bos taurus* and *Bos indicus* rather than pure *Bos indicus*.

Payne and Hodges [6] state that it is likely that many African cattle were *Bos taurus* before the rinderpest virus (RPV) epidemic of 1887–1897, which was reported to have more severely affected the taurine cattle populations of the East and South than the zebu populations. However, inferences about the types of cattle present in Africa until recent times are largely based on sparse depictions of humped (inferred as *Bos indicus* types) versus non-humped (inferred as *Bos taurus* type) cattle.

Our results show that, other than the pure African *Bos taurus* populations, all African indigenous cattle populations are admixtures of *Bos indicus* and African *Bos taurus*, with West African and Southern African populations showing lower *Bos indicus* admixture than East African populations consistent with recent studies by Pitt et al. [37] and Verdugo et al. [4]. The South African breeds, Africander and Tuli, show an even higher differentiation from East and West African breeds, as well as from other Sanga breeds based on *F*_*ST*_ values. This differentiation can be attributed at least in part to their admixture with European taurine breeds. Some indigenous breeds such as the Gobra show a surprisingly wide distribution of African taurine and indicine ancestry (Fig. 1a, Ndiaye et al. [38]), which likely reflects recent crossing to Guzerat (a pure *Bos indicus* breed) that was imported from Brazil into the region in which the Gobra samples used here were collected [39].

The PCA showed that the existing African *Bos taurus* populations and our reference European *Bos taurus* populations exhibit much greater diversity than the set of *Bos indicus* populations used in our analyses. Studies that included much larger samples of European *Bos taurus* cattle similarly showed greater diversity among European *Bos taurus* versus *Bos indicus* cattle (e.g. Decker et al., [40] and Mastrangelo et al., [41]. Our results indicate that the greater diversity of *Bos taurus* cattle extends to African *Bos taurus* in addition to European *Bos taurus*.

Populations with relatively high heterozygosity levels were Ankole-Watusi and N’Dama3, which show substantial admixture between populations with a large genetic distance (indigenous with European *Bos taurus,* and African *Bos taurus* with other indigenous, respectively). The distribution of indigenous breeds in the PC plots (Fig. 1a, b) and the results from Admixture (Table 1, *K* = 11) suggest a different African *Bos taurus* ancestry for West African versus East African indigenous.

The West African Bororo (sampled in Chad) clustered closely with the Fulani (sampled in Benin) and had very similar breed proportions, indicating that they likely form a single population. According to Grema et al. [42], Bororo is also known by several other names, including Red Fulani in West Africa supporting that Bororo and Fulani are one population with different names in different countries. Kuri is generally referred to as being an African *Bos taurus* breed [42], but our results show that Kuri is an admixture between African *Bos taurus* and *Bos indicus.* The tight clustering of the Kuri samples indicates that this is an old admixture rather than a recent hybridization.

Results for the Mpwapwa showed that this synthetic dual-purpose breed, first created about 60 years ago, aligns with its reported genetic history of 35% Sahiwal, 20% Red Sindhi, 35% East African zebu breeds, and 10% Ayrshire [43]. The Oulmes Zaer showed high heterozygosity consistent with its high European *Bos taurus* ancestry and being an admixture between African and European *Bos taurus*. Gautier and Naves [44], who analyzed the same Oulmes Zaer samples as here but with a very different set of 23 other breeds, also found that Oulmes Zaer was of hybrid origin between African and European *Bos taurus*. The rather dispersed cluster of the Oulmes Zaer samples in the PC plots suggests the possibility that the breed might have been deliberately created by the crossing of African and European *Bos taurus* cattle in relatively recent times.

Crossbreeding is widely used to achieve a suitable balance of productivity and adaptation in African smallholder dairy farming. According to the literature, the predominant exotic dairy breeds used in Kenya have been Holstein-Friesian, Ayrshire, Guernsey, and Jersey [45], and in Uganda Holstein-Friesian, with some use of Jersey, Guernsey, and Ayrshire [46]. In Ethiopia and Tanzania, the dominant exotic breeds are Holstein-Friesian and Jersey, and Holstein and Friesian, respectively [47, 48], while in Senegal Montbeliarde and Holstein are the dominant exotic breeds [49]. In the current study, the European dairy proportions found in East and West African crossbreds reflect the reported history of crossbreeding. Based on the PC plot (Fig. 4), the crossbred animals from Kenya, Ethiopia, and Tanzania distributed towards East African zebu, the dominant indigenous breed group in these countries, while in Uganda, they distributed toward the Ankole, which was the dominant indigenous breed in the areas sampled. These results are consistent with the findings of Weerasinghe et al. [13] and Strucken et al. [33] for the East African crossbred samples used here.

### Linkage disequilibrium and effective population size

Linkage disequilibrium is a measure of the non-random association of alleles at two or more loci that can be caused by selection and past and present population structure [50, 51]. As expected, the LD for all populations in our study was the highest (*r*^*2*^_adj_ > 0.2) for very short distances (about 10 Kb) with an exponential decline with increasing distance. The European reference dairy breeds showed a higher level of LD across all interval sizes compared to *Bos indicus* reference breeds and most African indigenous breeds (Figure S3), reflecting that intensive selection has caused relatively low effective population sizes generating high LD in the European dairy breeds [52, 53].

We found for all populations a steady, slow decline in *Ne* from 2000 generations until about 150 generations ago. Assuming a generation interval of about 6 years, this time range corresponds to about 12,000–900 years ago. The domestication of cattle has been dated to about 10,000 years ago [54, 55]. The *Bos indicus* reference breeds and all African indigenous populations, including the African *Bos taurus* breeds, show a substantially higher *Ne* 12,000 years ago (*Ne* = 5000 to 7000) than the European *Bos taurus* breeds (*Ne* = 2000 to 3500, Figure S4). Given the admixed *Bos indicus* and African *Bos taurus* ancestry of most African indigenous breeds, a high *Ne* pre-domestication is not unexpected, but the higher *Ne* of *Bos indicus* and African *Bos taurus* compared to European *Bos taurus* suggest that European *Bos taurus* was domesticated from a smaller population than African *Bos taurus* and *Bos indicus*. The *Ne* estimates obtained using *r*^2^_adj_ for recent generations for all indigenous populations are > 100, which should provide sufficient genetic diversity for the long-term survival of a population according to Meuwissen [56].

Estimates of *Ne* based on *r*^*2*^_adj_ stabilized or increased for most African indigenous breeds between about 200 generations and 30 to 10 generations ago (Figs. 8b, S5c and d). The process of domestication and stabilization of populations into genetically discrete populations could lead to lower effective population sizes over time, even if numbers of cattle overall were increasing with the spread of cattle farming. But as human populations and their associated livestock populations continued to expand, growth in numbers of cattle within each indigenous population could have outweighed downward pressure on *Ne*. An alternative explanation of the increase in *Ne* about 200 generations ago might be a period of hybridization between existing indigenous populations and/or between indigenous populations and *Bos indicus* cattle, following the migration of Bantu-speaking agropastoralists from eastern to southern Africa around 1500 years BP [57], and the second wave of *Bos indicus* cattle that are believed to have entered Africa with the migration of Arab peoples starting around 1500 to 1300 years BP.

Finally, a stabilization or increase in *Ne* might arise if the adjustment of *r*^*2*^ to account for sample size led to over-correction of *r*^*2*^ causing underestimation of *r*^2^_adj_ and hence overestimation of *Ne*. But in this case, the bias in *Ne* should be more pronounced for the most recent generations where *Ne* is estimated from very long-range estimates of LD, which have the lowest expectations of true *r*^*2*^ and hence *r*^2^_adj_ values that are closest to zero leading to high values of *Ne.* The expectation from theory is that very large sample sizes are required to avoid downward bias in the estimation of *Ne* [58]. Corbin et al. [59] confirmed this using simulation to show that before adjusting *r*^2^ values for sampling effect, increasing the sample size reduced the downward bias and improved the accuracy of estimates of *Ne* for recent generations. However, when *r*^2^ values were adjusted for sample size, the estimates of *Ne* were more stable. These authors suggested that the theoretical basis for models of variable *Ne* based on LD is not clear and has not been fully established. Therefore, the conservation decisions based on *Ne* from LD should be considered with caution.

## Conclusions

African indigenous cattle are genetically diverse due to historical and highly diverse admixture of *Bos indicus,* African *Bos taurus,* and European *Bos taurus.* This provides a great opportunity for future research and utilization, particularly for traits underlying adaptation to challenging environments. Our study shows that historical classifications of breeds only approximate the underlying genetic differences, with substantial overlap in the composition of breed groups historically thought to be distinct, such as Sanga and zebu. We also showed that African zebu cattle are not *Bos indicus* as continues to be stated in much of the published literature. The African continent is home to many more breeds than analyzed here, and it is to be hoped that more extensive application of molecular genetic assays will lead to the characterization of all the breeds of Africa yielding a comprehensive map of African cattle diversity, creating a platform for future utilization and characterization.

## Methods

### Animals and their sources

This study analyzed data on 4089 animals representing a diverse set of African indigenous and crossbred plus exotic cattle populations. In addition to the crossbreds, exotic reference breeds, one African synthetic breed, and one unclassified African indigenous breed were used, and 669 samples representing 33 breeds or populations within the three main African indigenous cattle breed groups of African *Bos taurus*, African zebu, and Sanga (Table 3). Data were obtained from several public-domain databases plus two projects run by the International Livestock Research Institute (ILRI) and collaborators, and the Dairy Genetics East Africa project (DGEA, Strucken et al. [33]). The main objective of DGEA was to identify the most suitable crossbred dairy cow genotypes for the range of dairy production systems and levels of production in Kenya, Uganda, Tanzania, and Ethiopia. The samples from Senegal [21, 39, 60] were collected as part of a study on the trade-offs of keeping different breed or cross-breed types of dairy cattle in smallholder systems in Senegal. A total of 644 samples were collected, with the breed or cross-breed type of the animal given by the farmer. The main breed types comprised indigenous breeds crosses between the indigenous breeds and the Guzerat (an imported *Bos indicus* dairy breed), and crosses between the indigenous breeds and other exotic dairy breeds, such as Montbeliarde and Holstein-Friesian.

**Table 3:** Animal populations, numbers, and sources

Breed	Breed group	Geographical location	Origin/Country	Number of animals	Genotype source
Ayrshire	EuB.t	Canada	Canada	20	CDN
Friesian	EuB.t	European	UK	20	SRUC
Guernsey	EuB.t	USA and UK	USA and UK	20	Bovine HapMap consortium [32]
Holstein	EuB.t	USA and NZ	USA and NZ	20	Bovine HapMap consortium [32]
Jersey	EuB.t	USA and NZ	USA and NZ	20	Bovine HapMap consortium [32]
Montbeliarde	EuB.t	European	France	20	Decker et al.*,* [40]
N’Dama	AfB.t	West African	Guinea	20	Bovine HapMap consortium [32]
N’Dama1	AfB.t	West African	Cote d’Ivoire	14	Decker et al. [40]
Lagune	AfB.t	West African	Benin	20	Decker et al. [40]
Somba	AfB.t	West African	Togo	20	Decker et al. [40]
Baoule	AfB.t	West African	Burkina Faso	20	Decker et al. [40]
Nelore	B.i	Brazil	Brazil	20	Bovine HapMap consortium [32]
Gir	B.i	Indian	Brazil	20	DGEA [33]
Sahiwal	B.i	Indian	Kenya	20	DGEA [33]
Guzerat*	B.i	Brazil	Senegal	8	Marshall et al. [39]
N’Dama2	AfB.t	West African	Southeast Burkina Faso	14	Decker et al. [40]
N’Dama3	AfB.t	West African	Southwest Burkina Faso	17	Decker et al. [40]
Lagunaire	AfB.t	West African	West Africa	5	Bovine HapMap consortium [32]
Africander	Sanga	Southern African	South Africa	4	Decker et al. [40]
Tuli	Sanga	Southern African	Botswana	4	Decker et al. [40]
Djakore	zebu	West African	Senegal	7	Marshall et al. [39]
Gobra	zebu	West African	Senegal	118	Marshall et al. [39]
Maure	zebu	West African	Senegal	12	Marshall et al. [39]
Gobara*Maure	zebu	West African	Senegal	10	Marshall et al. [39]
Bororo	zebu	West African	Chad	20	Decker et al. [40]
Fulani	zebu	West African	Benin	20	Decker et al. [40]
Kuri	AfB.t	West African	Chad	20	Decker et al. [40]
Borgou	zebu	West African	Benin	20	Decker et al. [40]
Ankole	Sanga	East African	Uganda	20	DGEA [33]
Ankole-Watusi	Sanga	East African	Ruanda	5	Decker et al. [40]
SEAZ	zebu	East African	Kenya	21	DGEA [33]
Madagascar-zebu	zebu	Madagascar	Madagascar	20	Decker et al. [40]
Danakil-Harar	zebu	East African	Ethiopia	30	DGEA [33]
Begait-Barka	zebu	East African	Ethiopia	27	DGEA [33]
Boran	zebu	East African	Ethiopia	28	DGEA [33]
Boran1	zebu	East African	Ethiopia	20	Decker et al. [40]
Boran	zebu	East African	Kenya	28	DGEA [33]
Fogera	zebu	East African	Ethiopia	28	DGEA [33]
Iringa-Red	zebu	East African	Tanzania	11	DGEA [33]
Singida-White	zebu	East African	Tanzania	22	DGEA [33]
Central Highland	zebu	East African	Ethiopia	9	DGEA [33]
Sheko	Sanga	East African	Ethiopia	17	Decker et al. [40]
Sheko1	Sanga	East African	Ethiopia	18	Bovine HapMap consortium [32]
Mpwapwa	Synthetic	East African	Tanzania	20	DGEA [33]
Oulmes Zaer	Indigenous	North African	Morocco	19	Decker et al. [40]
Kenyan crossbred	Crossbred	East African	Kenya	1378	DGEA [33]
Uganda crossbred	Crossbred	East African	Uganda	555	DGEA [33]
Ethiopia crossbred	Crossbred	East African	Ethiopia	545	DGEA [33]
Tanzania crossbred	Crossbred	East African	Tanzania	462	DGEA [33]
Senegal crossbreed	Crossbred	West African	Senegal	253	Marshall et al. [39]
**Total**				**4089**	

*Guzerat was imported from Brazil; *EuB.t* European *Bos taurus*, *AfB.t* African *Bos taurus*, *B.i Bos indicus*, *SEAZ* Small East African Zebu, *USA* United States of America, *UK* United Kingdom, *NZ* New Zealand, *SRUC* Scottish Rural University College, *CDN* Canadian Dairy Network; Crossbreds are crosses between EuB.t. dairy breeds and local indigenous breeds; synthetic is a breed created by the deliberate crossing of several founder breeds; Indigenous here refers to a single African breed that does not fit into the other classifications

*Bos indicus* and European *Bos taurus* breeds were included to represent the major known anchor points of global cattle diversity against which African cattle diversity can be assessed. The European *Bos taurus* breeds were chosen because these breeds are known to have contributed to the crossbred dairy populations included in our study, allowing clearer interpretation of results than inclusion of other breeds or a larger sample of breeds. The reference breeds included five African *Bos taurus* (N’Dama, N’Dama1, Lagune, Baoule, and Somba), four *Bos indicus* (Nelore, Sahiwal, Gir, and Guzerat), and six European *Bos taurus* dairy breeds (Guernsey, Holstein, Jersey, Canadian Ayrshire, British Friesian, and Montbeliarde). The reference data were obtained from the Bovine HapMap Consortium [32], Decker et al. [40], the Scottish Rural University College (SRUC), and the Canadian Dairy Network (CDN, Table 3).

### Genotyping and quality control

The Senegal animals were genotyped using the Illumina BovineSNP50v2 BeadChip array (Illumina Inc., San Diego, CA, USA) comprising 54,609 SNPs. Quality control was carried out using the GenABEL package [61] in R Core Team [62]. Autosomal SNPs were retained, and SNPs and animals with call-rates lower than 90% were excluded. No threshold criteria for minor allele frequency (MAF) or Hardy-Weinberg-Equilibrium (HWE) were applied because low MAF SNPs can provide powerful information for breed differentiation analyses and HWE is not expected to yield reasonable results for some populations due to admixture and small population size. A total of 45,809 SNPs and 628 animals remained after quality control.

Samples from the DGEA project were genotyped with the Illumina BovineHD Beadchip (Illumina Inc., San Diego, CA, USA) and sourced from Strucken et al. [33]. The DGEA data of 777 k SNPs was quality controlled according to similar thresholds as described in West Africa. Approximately 735 k SNPs remained after QC. The 777 k data from the Bovine HapMap Consortium, SRUC, and CDN were also supplied quality controlled. The 50 k data from Decker et al. [40] were genotyped with the Illumina BovineSNP50 BeadChip array (Illumina Inc., San Diego, CA, USA), and supplied post quality control. Merging and retaining only those SNPs present across all datasets resulted in a subset of 38,556 SNPs.

### Analysis of the genetic structure

Principal component analyses were performed using a GRM to define the covariance between animals. Two separate PCA were conducted: 1) where the GRM was based on all cattle populations; 2) the GRM was built using only African taurine breeds. The second GRM was used to evaluate the genetic structure and diversity among the African taurine breeds to select the African *Bos taurus* reference populations to be used in the Admixture analysis. The GRMs were constructed according to the first method of VanRaden [63]. Genotypes were recorded as allele counts of 0, 1, and 2, which were converted to − 1, 0, 1 to centre the genotype matrix **M**. Missing genotypes were replaced with the average allele frequencies across all animals for each given SNP. The GRM was then calculated as:

$$ GRM= ZZ^{\prime }/d $$

where the scaling parameter d was:

$$ d=2\ast \sum \left({p}_i\ast \left(1-{p}_i\right)\right) $$

The centred genotype matrix (**Z**) was constructed by subtracting the **P** matrix from the genotype matrix **M**, where **P** = 2 ∗ (*p*_*i*_ − 0.5), and *p*_*i*_ is the allele frequency at locus *i*.

To investigate the genetic admixture of the indigenous and crossbred cattle populations, a maximum likelihood model implemented in the software ADMIXTURE 1.23 [64] was applied. Supervised analyses were used because unsupervised analyses become unstable as *K* is increased, i.e., individual true ancestry proportions are low, and estimates become uninterpretable and often over-predicted [65]. Also, prior literature [33, 66] and our analyses have previously demonstrated that there are three ancestral populations of African cattle (African *Bos taurus*, European *Bos taurus,* and *Bos indicus*), and the use of supervised analyses allows clear dissection of these contributions. A pooled sample of *Bos indicus* reference (indicine) was created with eight animals per breed because of the four indicine reference breeds clustered very closely together in the PCA (Figure S1a). The African taurine samples showed larger breed differences compared to the indicine reference breeds (Figure S1b) and were therefore considered as separate reference populations. ADMIXTURE was used in 3 alternative supervised analyses where the number of reference breeds was set to 7, 11, and 12. The assumed ancestral populations in Model 1 (*K* = 7) were: N’Dama (African taurine), Indicine (pooled *Bos indicus* samples), Ayrshire, Friesian, Guernsey, Holstein, and Jersey. In Model 2 (*K* = 11), N’Dama1, Lagune, Baoule, and Somba were added as African taurine reference breeds. In Model 3 (*K* = 12), Montbeliarde was added as another European dairy breed, because Montbeliarde has been used for crossbreeding to indigenous cattle in Senegal.

### Genetic relatedness and differentiation

Pairwise *F*_*ST*_ values were calculated according to Weir and Cockerham [67], where *F*_*ST*_ is defined as the genetic variance between populations expressed as a proportion of the total genetic variance. The degree of inbreeding was inferred from the *F*_*IS*_ coefficient calculated according to Nei [68], where the *F*_*IS*_ is defined as one minus the observed proportion of heterozygotes divided by the expected proportion of heterozygotes. To explore genetic differentiation among breeds, *F*_*ST*_ values were visualized in a heatmap, and the complete-linkage method was used for hierarchical clustering as provided in the R package “pheatmap” [69].

### The extent of linkage disequilibrium and effective population size in pure breeds

The extent and magnitude of LD within different breeds were determined using *r*^*2*^ between phased alleles of pairwise SNP loci. The genotypes were phased using Eagle v2.4 [70], and the LD coefficients were calculated with VCFtools v0.1.15 [71]. The *r*^2^ was estimated separately for each breed between all pairs of SNPs with a distance of up to 50 Mb using markers with minor allele frequency ≥ 5% according to the Hill and Roberson formula [72]:

$$ {r}^2=\frac{D^2}{f(A)\times f(a)\times f(B)\times f(b)} $$

where,

$$ D=f(AB)-f(A)f(B) $$

where *f(AB)* is the observed frequency of haplotype AB [73], while *f(A)*, *f(a)*, *f(B)* and *f(b)* are observed frequencies of alleles *A*, *a*, *B*, and *b*, respectively. For the purpose of graphical display, the distance of pair-wise LD was binned into 10 Kb intervals up to 1 Mb.

The *r*^*2*^ values combined with marker distances were used to estimate the approximate *Ne* at a given time point in the past, assuming a model without mutation, and using the formula of Sved [74]:

$$ Ne=\left(\frac{1}{4c}\right)\left(\frac{1}{r^2}-1\right) $$

where *Ne* is the effective population size, and *c* the marker distance in Morgans assuming 1 M = 100Mbp. The time (number of generations ago) at which *Ne* was estimated as 1/2*c* [52]. For estimating the effective population size, the data were grouped into 80 bins of 25 Kb for SNPs that were up to 2 Mb while, for a distance of more than 2 Mb up to 50 Mb, the number of previous generations (25 to 1) was selected, and the appropriate range of c was calculated. The binning process was designed to ensure sufficient SNP pairs within each bin and to obtain a representative average *r*^2^ across the autosomes. This model does not account for sampling bias of *r*^*2*^ due to small population sizes. We extended the model to account for sample size according to Weir and Hill [75]:

$$ Ne=\left(\frac{1}{4c}\right)\left(\frac{1}{{r^2}_{adj}}-1\right) $$

where ${r^2}_{adj}={r}^2-\frac{1}{\left(2\ast N\right)}$ with N being the sample size. To reduce the sampling error and bias in estimates of *Ne*, *Ne* was estimated only for populations that have a sample size ≥20. The inclusion of related animals in the sample can cause an upwards bias of *Ne* (particularly recent *Ne*). Estimates of *Ne* presented in this paper are from samples that exclude animals that have a GRM relationship > 0.2 with one or more animals.

## Supplementary Information

**Additional file 1: Figure S1.** PC1 vs. PC2 when using the whole dataset. (a) Expanded plot *Bos indicus* breeds section. (b) Expanded plot African *Bos taurus* breeds section.

**Additional file 2: Figure S2.** PCA using African *Bos taurus* populations (a) Plot of PC1 vs PC2. (b) PC1 vs PC3.

**Additional file 3: Figure S3.** Decline of linkage disequilibrium (*r*^*2*^) with physical distance (kbp). (a) *Bos taurus* dairy breeds. (b) *Bos indicus* reference breeds. (c) East and South African indigenous breeds. (d) West African indigenous breeds.

**Additional file 4: Figure S4.** Effective population size over past generations using *r*^2^ (log-scaled) for (a) *Bos taurus* dairy breeds, (b) *Bos indicus* breeds, (c) East and Southern African indigenous breeds, and (d) West African indigenous breeds.

**Additional file 5: Figure S5.** Effective population size over past generations using *r*^2^_adj_ (log-scaled) for (a) *Bos taurus* dairy breeds, (b) *Bos indicus* breeds, (c) East and Southern African indigenous breeds, and (d) West African indigenous breeds.

**Additional file 6: Table S1.** Estimates (±SD) of *F*_*IS*_ (diagonal) within the breeds and pairwise *F*_*ST*_ (above diagonal) values between the breeds.

## Data Availability

Data were sourced from a variety of public domain and privately held databases as detailed in the paper. In most cases, the data held privately is available on request to the institution owning the data.
