# Supplementary figures and images for: The patterns of admixture, divergence, and ancestry of African cattle populations determined from genome-wide SNP data
Source: BMC Genomics. 2020 Dec 7;21:869. doi: 10.1186/s12864-020-07270-x (PMC7720612; doi:10.1186/s12864-020-07270-x)

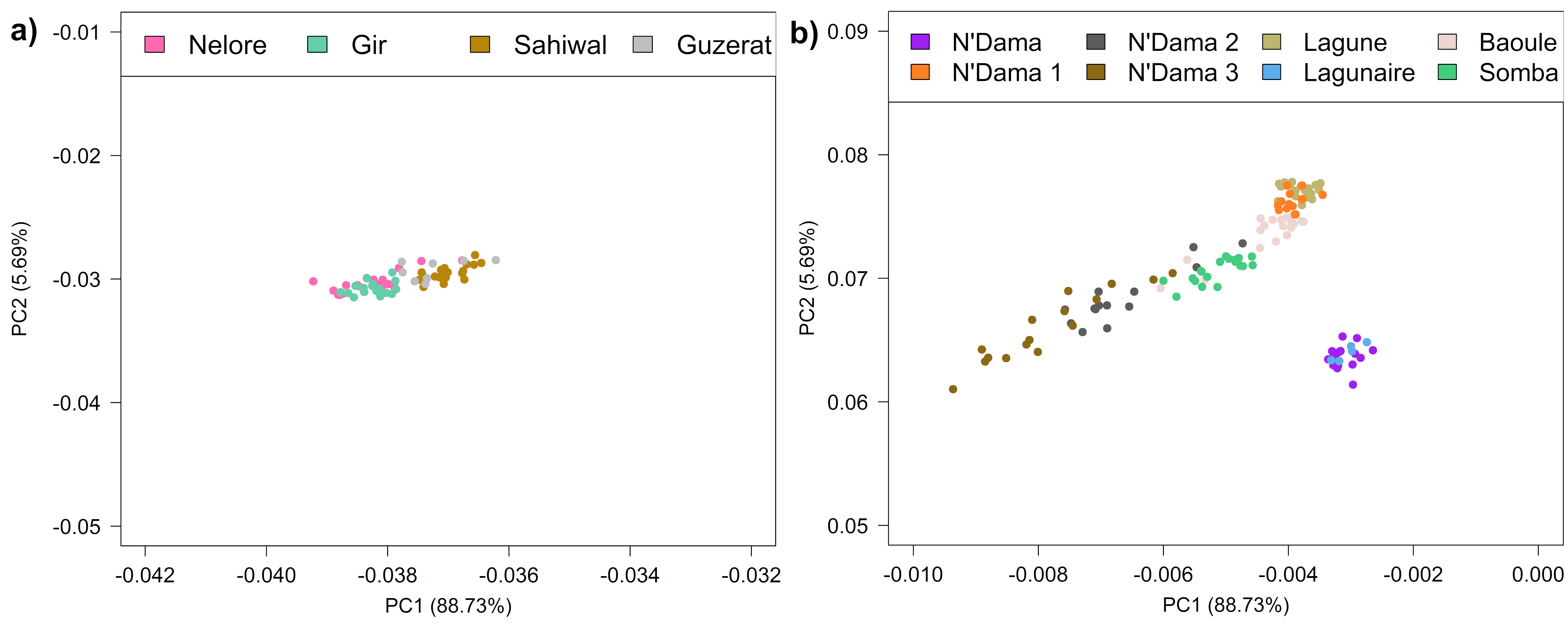

Supplement: Supplementary file 1 — Additional file 1: Figure S1. PC1 vs. PC2 when using the whole dataset. (a) Expanded plot Bos indicus breeds section. (b) Expanded plot African Bos taurus breeds section. [file 12864_2020_7270_MOESM1_ESM.tiff]

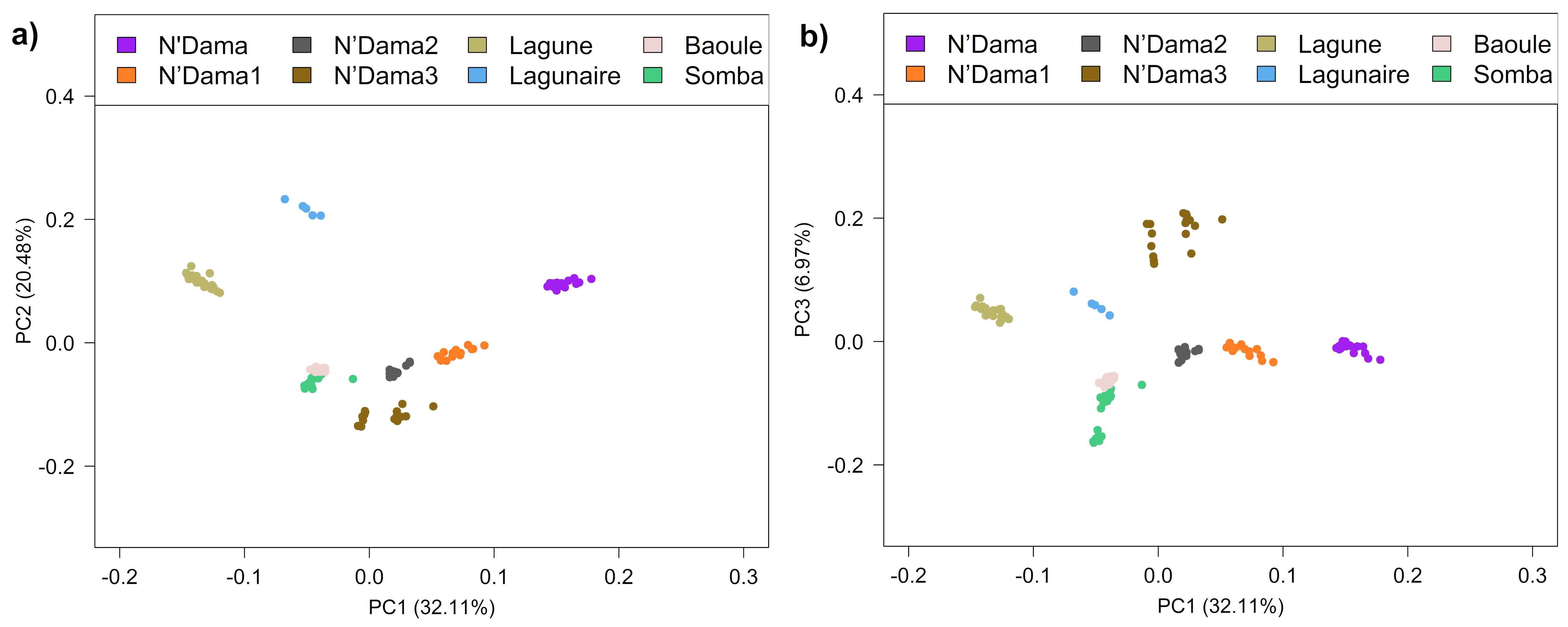

Supplement: Supplementary file 2 — Additional file 2: Figure S2. PCA using African Bos taurus populations (a) Plot of PC1 vs PC2. (b) PC1 vs PC3. [file 12864_2020_7270_MOESM2_ESM.tiff]

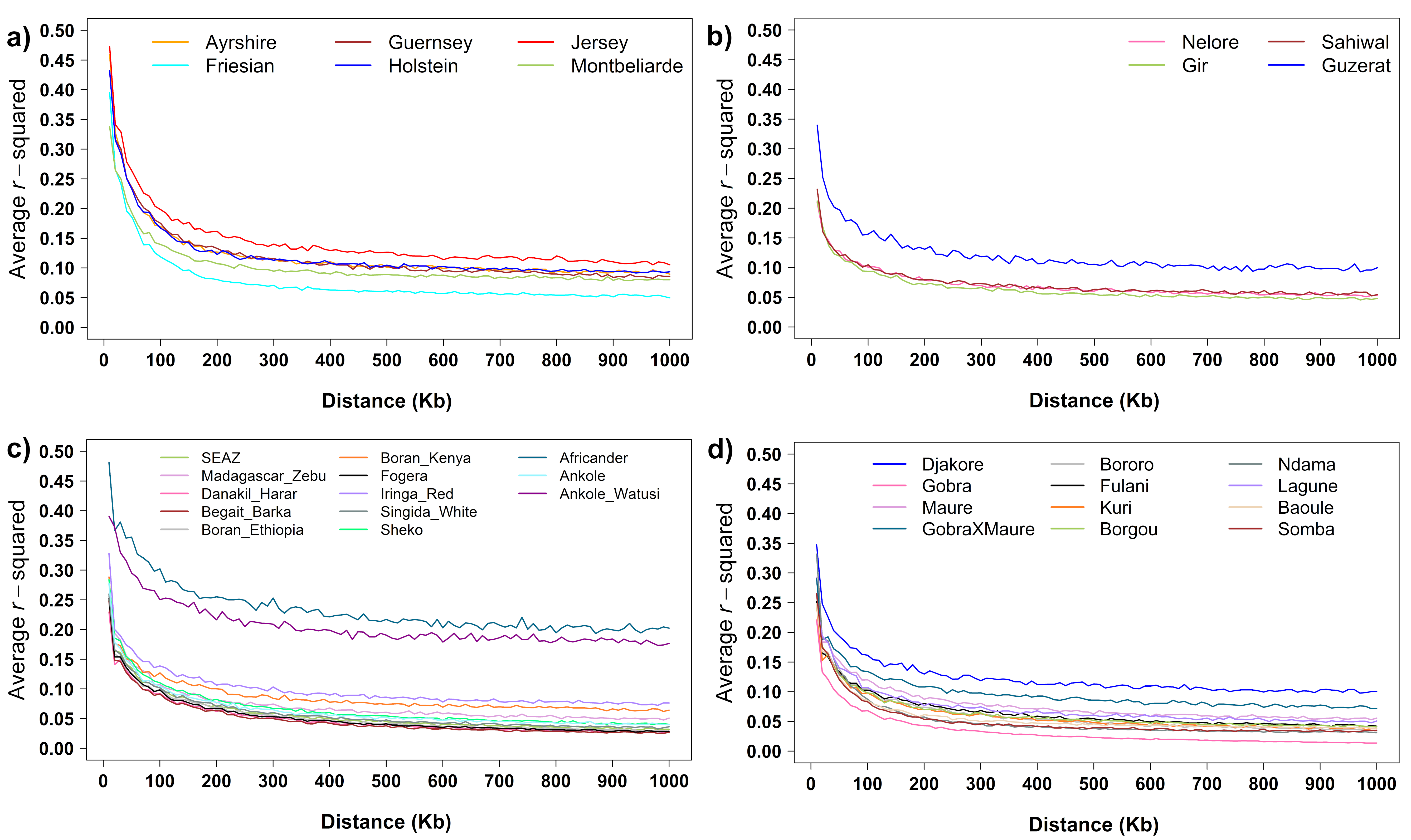

Supplement: Supplementary file 3 — Additional file 3: Figure S3. Decline of linkage disequilibrium (r2) with physical distance (kbp). (a) Bos taurus dairy breeds. (b) Bos indicus reference breeds. (c) East and South African indigenous breeds. (d) West African indigenous breeds. [file 12864_2020_7270_MOESM3_ESM.tiff]

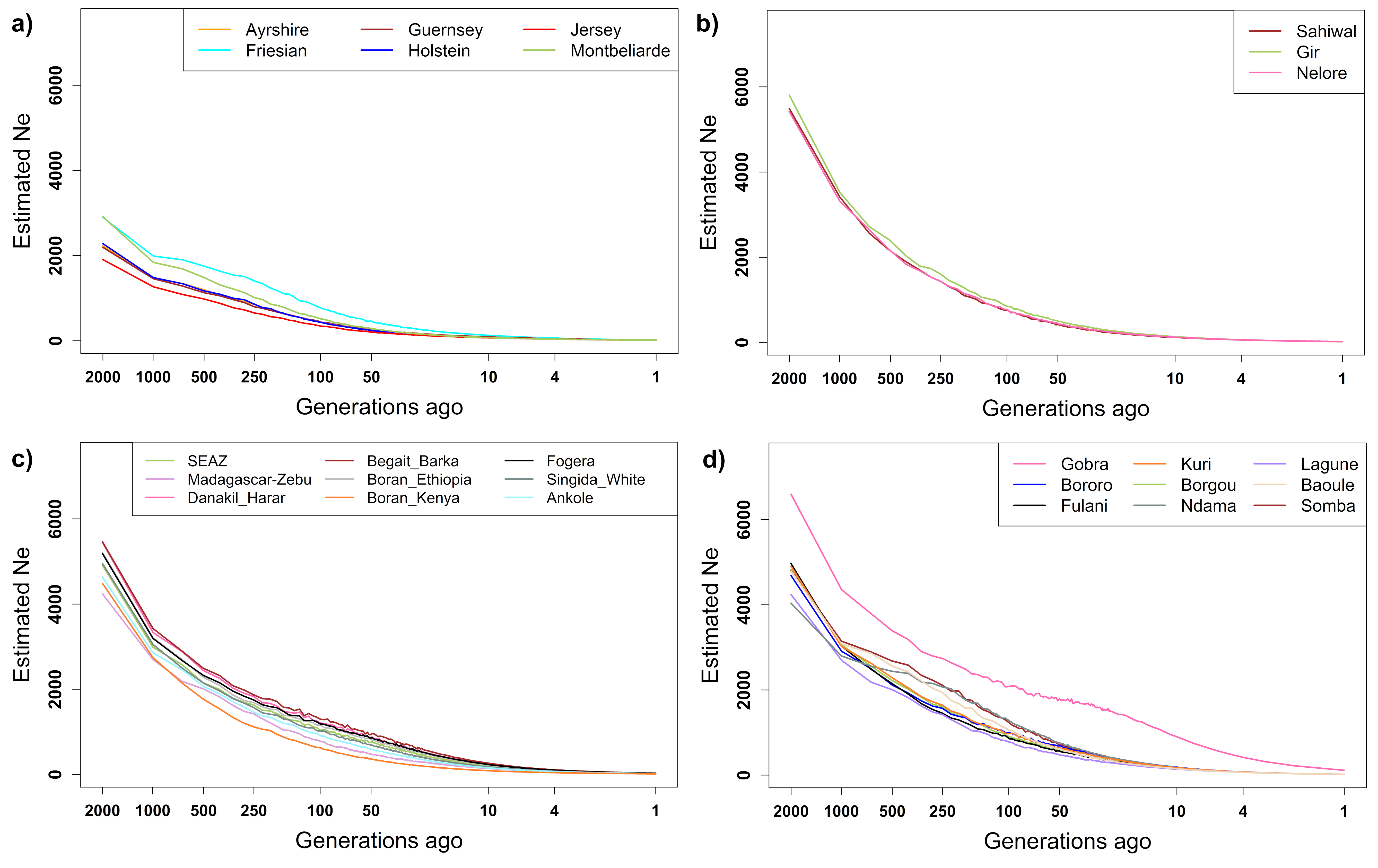

Supplement: Supplementary file 4 — Additional file 4: Figure S4. Effective population size over past generations using r2 (log-scaled) for (a) Bos taurus dairy breeds, (b) Bos indicus breeds, (c) East and Southern African indigenous breeds, and (d) West African indigenous breeds. [file 12864_2020_7270_MOESM4_ESM.tiff]

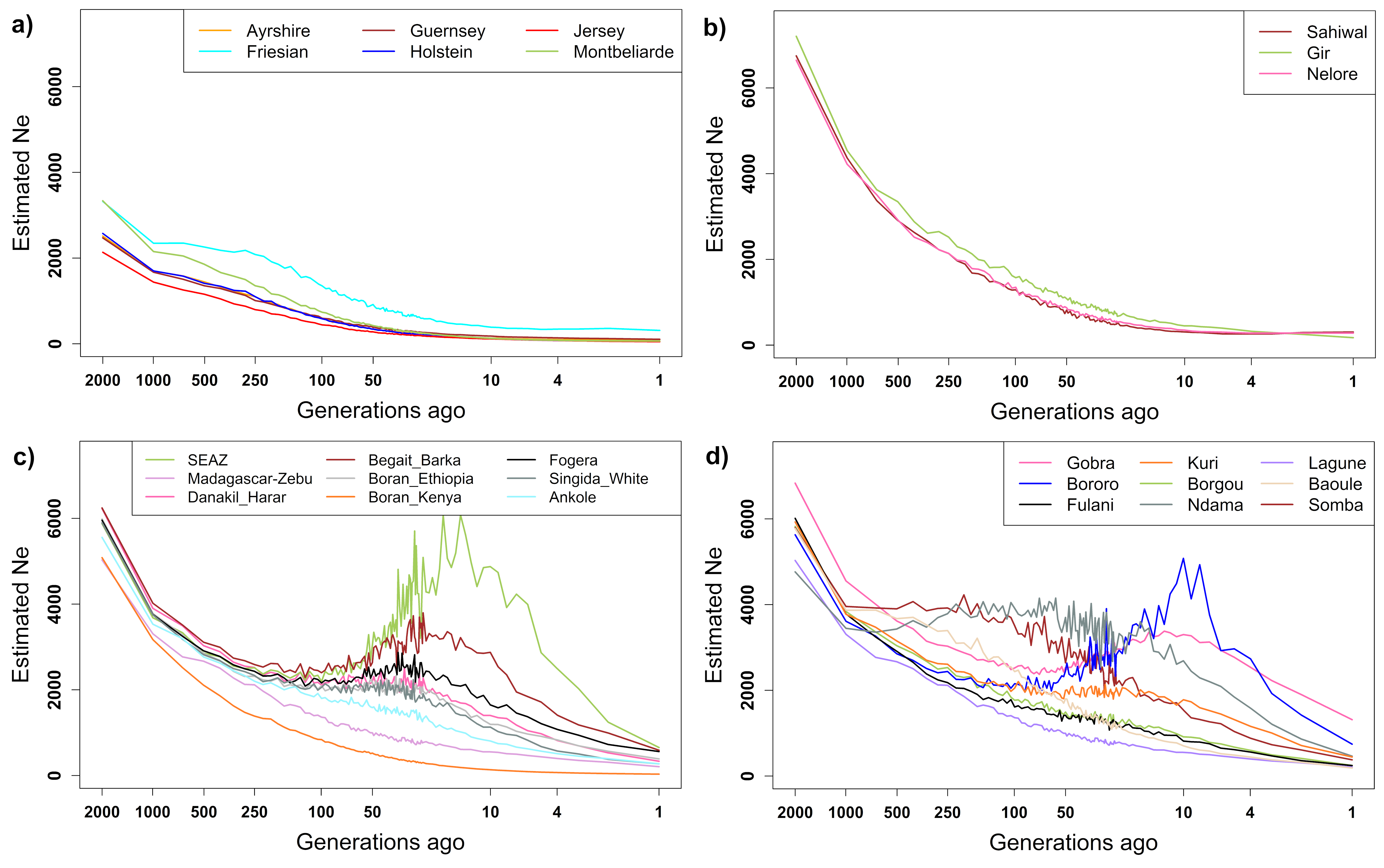

Supplement: Supplementary file 5 — Additional file 5: Figure S5. Effective population size over past generations using r2adj (log-scaled) for (a) Bos taurus dairy breeds, (b) Bos indicus breeds, (c) East and Southern African indigenous breeds, and (d) West African indigenous breeds. [file 12864_2020_7270_MOESM5_ESM.tiff]
